# Potential of asphericity as a novel diagnostic parameter in the evaluation of patients with ^68^Ga-PSMA-HBED-CC PET-positive prostate cancer lesions

**DOI:** 10.1186/s13550-017-0333-9

**Published:** 2017-10-23

**Authors:** Sebastian Meißner, Jan-Carlo Janssen, Vikas Prasad, Winfried Brenner, Gerd Diederichs, Bernd Hamm, Frank Hofheinz, Marcus R. Makowski

**Affiliations:** 1 Department of Radiology, Charité Universitätsmedizin Berlin, Charitéplatz 1, 10117 Berlin, Germany; 2 Department of Nuclear Medicine, Charité, Charitéplatz 1, 10117 Berlin, Germany; 3 Helmholtz Zentrum Dresden-Rossendorf, Bautzner Landstraße 400, 01328 Dresden, Germany

**Keywords:** Prostatic neoplasms, Positron emission tomography computed tomography, Gleason score, Asphericity, Histopathology

## Abstract

**Background:**

The aim of this study was to evaluate the diagnostic value of the asphericity (ASP) as a novel quantitative parameter, reflecting the spatial heterogeneity of tracer uptake, in the staging process of patients with ^68^Ga-PSMA-HBED-CC positron emission tomography (PET)-positive prostate cancer (PC).

In this study, 37 patients (median age 72 years, range 52–82 years) with newly diagnosed PC, who received a ^68^Ga-PSMA-HBED-CC PET fused with computed tomography (^68^Ga-PSMA-PET/CT), a magnetic resonance imaging (MRI) of the prostate, and a core needle biopsy (within 74.2 ± 80.2 days) with an available Gleason score (GSc) were extracted from the local database. The ASP and the viable tumor volume (VTV) was calculated using the rover software (ABX GmbH, Radeberg, Germany), a segmentation tool for automated tumor volume delineation. Additionally, parameters including total lesion binding rate (TLB), maximum, mean and peak standardized uptake value (SUVmax/mean/peak), prostate-specific antigen (PSA), D’Amico classification, and prostate imaging reporting and data system (PI-RADS) were analyzed.

**Results:**

The ASP mean differed significantly (*p* ≤ 0.05) between the different GSc groups: GSc 6–7: 11.9 ± 4.8%, GSc 8: 25.5 ± 4.8%, GSc 9–10: 33.3 ± 6.8%. A significant correlation between ASP and GSc (rho = 0.88; CI 0.78–0.94; *p* < 0.05) was measured. The ASP enabled an independent (*p* > 0.05) prediction of the GSc. A moderate correlation was measured between ASP and the D’Amico classification (rho = 0.6; CI 0.32–0.78; *p* < 0.05). The VTV showed a moderate correlation with the SUVmax (rho = 0.58; CI 0.32–0.76; *p* < 0.05) and the GSc (rho = 0.51; CI 0.23–0.72; *p* < 0.05).

**Conclusion:**

The asphericity in ^68^Ga-PSMA-PET could represent a promising novel quantitative parameter for an improved non-invasive tumor staging of patients with PC.

## Background

Prostate cancer (PC) is the most frequent cancer entity diagnosed in men in the western world with the second highest overall mortality [1]. PC is responsible for up to 8% of all cancer-related deaths in males, resulting in the fourth leading cause of cancer-related death in both sexes [1]. The prostate-specific antigen (PSA) blood level is currently the reference standard for PC screening of the population. With a risk reduction of 1.07 deaths per 1000 cases, PSA screening was shown to reduce PC-associated mortality up to 21% [2]. However, it is not fully elucidated to which extent PSA reduces the all-cause PC-associated mortality [2]. Besides the positive effects of PSA screening, PSA testing can also be associated with an overdiagnosis of up to 23–42% [3, 4]. While advances, especially in the non-invasive local staging of patients with primary PC, have been made using magnetic resonance imaging (MRI) in recent years, this technique is still associated with limitations regarding the grading of the primary tumor [5].

To improve the local and whole body staging of patients with PC, different nuclear imaging probes were evaluated in recent years [6]. These include tracers based on N-methyl-^11^C-choline (^11^C-C) or ^18^F-fluoromethyl-[1,2-2H4]choline (^18^F-C), ^11^C-acetate, 2-amino-4-^11^C-methylsulfanyl-butanoic acid, 1-S-methyl-^11^C-methionine (^11^C-MET), ^18^F-fluorodihydrotestosterone (^18^F-FDHT), and 2-deoxy-2-^18^F-fluoro-D-glucose (^18^F-FDG) [7]. These tracers are not commonly used in clinical practice, as they have a limited sensitivity and specificity in staging of PC [6–11]. A recently introduced novel tracer, the ^68^Ga-N,N-bis[2-hydroxy-5-(carboxyethyl)benzyl]-ethylendiamine-N,N diacetic acid-labeled prostate-specific membrane antigen (PSMA) inhibitor Glu-NH-CO-NH-Lys(Ahx)-HBED-CC (^68^Ga-PSMA-HBED-CC), has shown promise to improve the local and whole-body staging of patients with PC [12]. ^68^Ga-PSMA-HBED-CC demonstrates a strong affinity to the prostate-specific membrane antigen and showed promising results in diagnosing recurrent PC, even at low PSA blood levels, and was found to be superior to standard routine imaging for preoperative lymph node staging in primary PC patients [13, 14].

Most current studies, which investigate positron emission tomography (PET) tracer uptake in different malignancies, rely on the maximum standardized uptake value (SUVmax) as a quantitative parameter for the characterization of the binding/uptake of the tracer. However, it is increasingly recognized that not only the overall uptake or metabolism, but also the heterogeneity of the tracer uptake, plays a role in the characterization of malignancies [15–18]. The quantification of the heterogeneity of tracer uptake could represent a promising novel parameter for an improved characterization of tumor heterogeneiety and therefore the malignancy of tumors [19]. In this context, a novel parameter—the asphericity (ASP)—was recently introduced [15]. The ASP describes the non-spherical shape of a tumor, compared to a sphere with the same volume. Initially, the ASP was used to quantify the spatial irregularity of the metabolic tumor volume (MTV) in ^18^F-FDG-PET [15, 18–21]. Most tumors are genetically and histopathologically heterogenic and further dedifferentiation and infiltration is often associated with poorer prognosis [15]. Previous studies demonstrated the potential of the ASP for an improved tumor staging [15, 19].

The aim of this study was to test the diagnostic value of the ASP in the staging process of patients with ^68^Ga-PSMA-HBED-CC-PET (^68^Ga-PSMA-PET)-positive PC.

## Methods

### Study population

This retrospective study was approved by the local ethics review board. The local database was screened for patients, who received a ^68^Ga-PSMA-PET combined with computed tomography (CT) and a 3 T MRI of the prostate within 110 days for staging of suspected primary PC. MRI was used as reference standard for the definition of the primary tumor and to evaluate our delineation process. The proximity of the ^68^Ga-PSMA PET/CT to the MRI was a requirement to ensure that the lesion which was evaluated in ^68^Ga-PSMA PET reflects the primary PC lesion. We extracted 691 patients from our imaging database, who underwent ^68^Ga-PSMA-PET/CT in-between Oct. 01, 2013 and Feb. 01, 2017. MRI data of the prostate was available in 169 cases, excluding 522 cases without a MRI of the prostate. In our institution, 3 T MRI has established itself as the routine MR examination for the evaluation of patients with prostate cancer. Twenty-six cases were excluded as only 1.5 T MRI datasets were available. Ninety patients were excluded as the delay between ^68^Ga-PSMA-PET/CT and 3 T MRI was more than 110 days. All PSMA-positive lesions had to be confirmed through core needle biopsy, which was not available for this study in 16 patients. The final cohort of 37 patients had a mean age of 71.3 ± 7.5 years and received both scans within 50.2 ± 32.5 days. Core needle biopsy and ^68^Ga-PSMA-PET/CT were performed within 74.2 ± 80.2 days. A detailed overview regarding the patient characteristics are summarized in Table 1.

**Table 1:** Characteristics of study collective

	Mean	SD	Median	Range
Age (years)	71.3	7.5	72	52–82
Days PSMA-PET to MRI	50.2	32.5	42	1–110
PI-RADS score	4.7	0.6	5	3–5
Days GSc to PSMA-PET	74.2	80.2	46	3–299
Gleason score	7.9	1.1	8	6–10
Days PSA to PSMA-PET	16.2	25.6	3	0–84
PSA (ng/ml)	17.7	21.5	11	0.23–116
VTV (cm^3^)	12.3	11.3	9	0.8–54.1

This table summarizes the main characteristics of the patients investigated in this study. This included the age of the patients, the PI-RADS and Gleason score, PSA blood level, and the viable tumor volume as well as the time difference between PSMA-PET and MRI, between Gleason score and PSMA-PET, between PSA blood level sampling date and PSMA-PET. Data are given in means, standard deviations, medians, and ranges. *Abbreviations: GSc* Gleason score, *PSA* prostate-specific antigen blood level, *PSMA* prostate-specific membrane antigen, *PET* positron emission tomography, *MRI* magnetic resonance imaging, *PI-RADS* prostate imaging reporting and data system, *VTV* viable tumor volume

### Positron emission tomography tracer

Elution of ^68^Ga was performed using a standard ^68^Ge/^68^Ga generator (Eckert and Ziegler) [22]. Next, PSMA-HBED-CC (ABX GmbH, Radeberg, Germany) was labeled with ^68^Ga [23, 24].

### Imaging protocol

PET/CT imaging was performed 75.4 ± 27.5 min after intravenous injection of 122.4 ± 19.7 MBq of [^68^Ga]-PSMA-HBED-CC. A 3D acquisition mode was used on a Gemini TF 16 Astonish PET/CT scanner (Philips Medical Systems) [25]. Default parameter settings were used in the system software to reconstruct the transaxial slices (144 × 144 voxels, 4 mm^3^). Immediately before the PET scan, a low-dose CT was acquired for attenuation correction (120 kVp, 30 mAs) and anatomical mapping.

### Asphericity

Definition of ASP:

$$ \mathrm{ASP}={100}^{\ast}\left(\sqrt[3]{H}-1\right)\kern0.5em \mathrm{with}\kern0.5em H=\frac{1\kern0.5em {S}^3}{36\uppi {V}^2} $$

ASP is defined as a marker for non-spherical tumor volumes. A sphere has the smallest possible surface *S* for a given volume *V* for which ASP = 0 by definition [15]. For non-spherical lesions, ASP > 0 provides a quantitative measure for deviation of spherical shape. For example, an ASP of 30% means a 30% larger surface than a sphere with the same volume [19]. A detailed description of the definition was published earlier [15].

### Imaging analysis

**Fig. 1:**
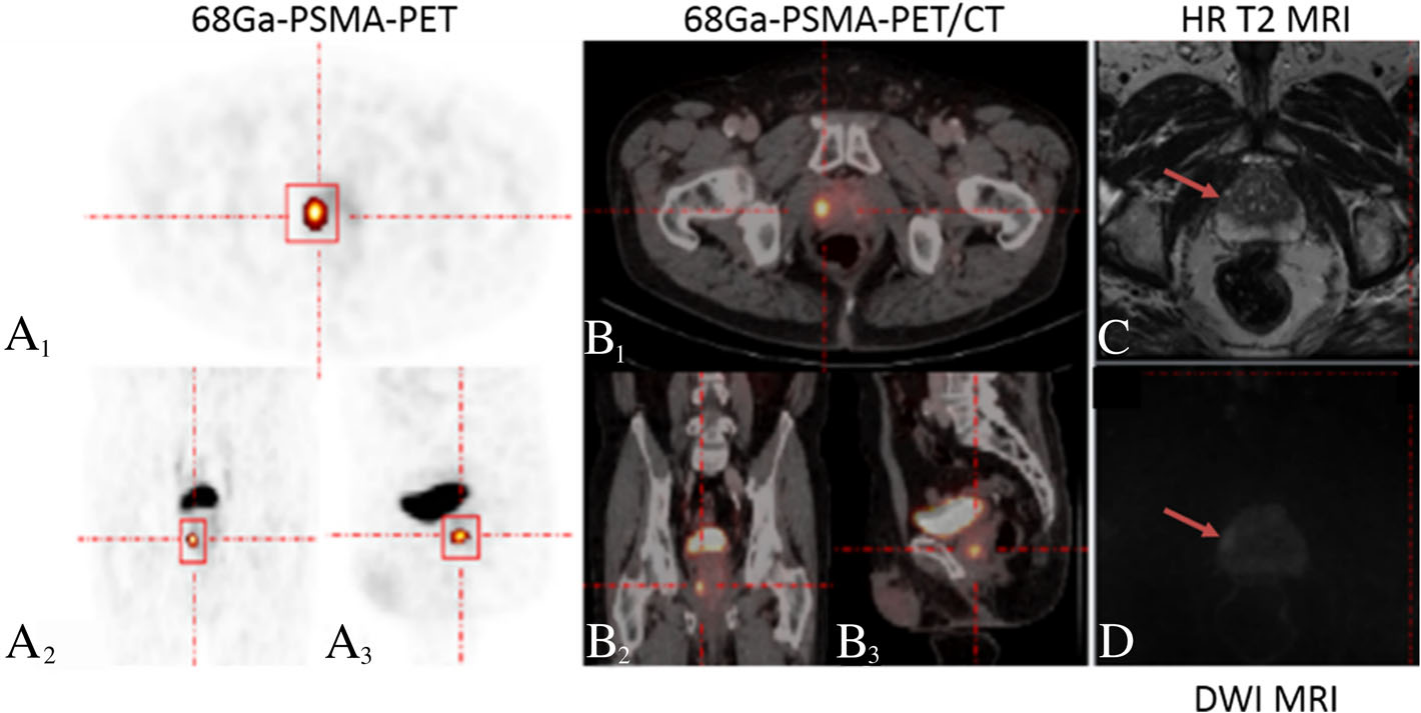
Example of tumor heterogeneity for the asphericity assessment in a patient diagnosed with a Gleason score 6 prostate cancer. **A**–**D** Example of a patient diagnosed with a Gleason score 6 prostate cancer in the right peripheral zone of the apex of the prostate. **A**, **B** Corresponding orientations (transversal (**A**_**1**_), coronal (**A**_**2**_), and sagittal (**A**_**3**_)) are shown for 68Gallium-labeled prostate-specific membrane antigen positron emission tomography, fused with computed tomography (**B**_**1–3**_). Based on positron emission tomography, an avid tumor was delineated in all three planes using the rover software. **C**, **D** Corresponding magnetic resonance imaging in the high-resolution T2 turbo spin echo (**C**) and diffusion-weighted images (**D**) in axial images confirm the presence of the malignant lesion at the respective location. HR: high-resolution, DWI: diffusion-weighted imaging

**Fig. 2:**
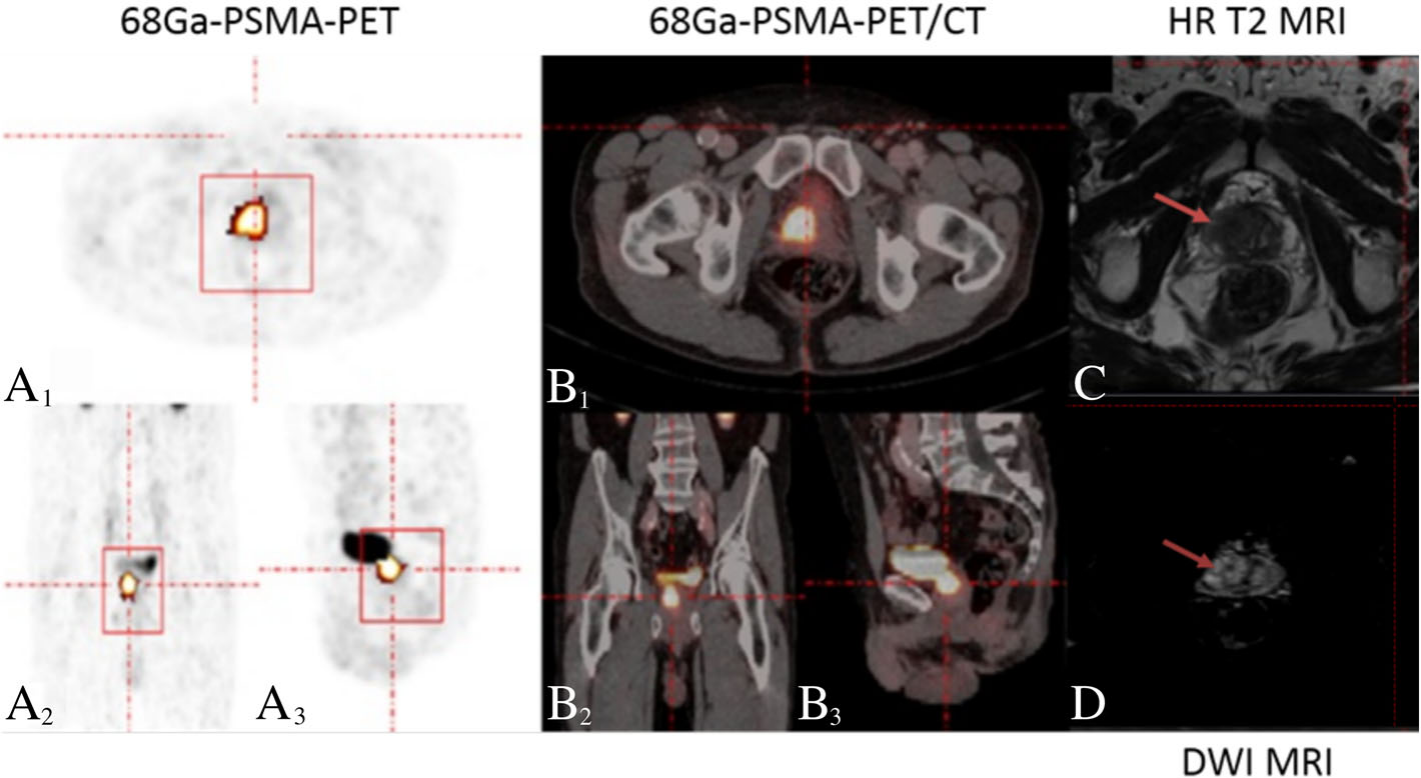
Example of tumor heterogeneity for the asphericity assessment in a patient diagnosed with a Gleason score 9 prostate cancer. **A**–**D** Example of a patient diagnosed with a Gleason score 9 prostate cancer in the in the right peripheral zone of the midgland of the prostate. **A**, **B** Corresponding orientations (transversal (**A**_**1**_), coronal (**A**_**2**_), and sagittal (**A**_**3**_)) are shown for 68Gallium-labeled prostate-specific membrane antigen positron emission tomography, fused with computed tomography (**B**_**1–3**_). Based on positron emission tomography, an avid heterogeneous tumor was delineated in all three planes using the rover software. **C**, **D** Corresponding magnetic resonance imaging in T2 turbo spin echo (**C**) and diffusion-weighted images (**D**) in axial images confirm the presence of the maligne lesion at the respective location. HR: high-resolution, DWI: diffusion-weighted imaging

The program Visage 7.1 (Visage Imaging) was used prior to the delineation process for evaluation of the ^68^Ga-PSMA-PET/CT and the MRI data. In case of multiple foci or lesions with unclear borders in PET, a simultaneous evaluation of the MRI data in Visage 7.1 supported the detection/delineation of the primary PC lesion. Following the detection of the primary lesion within the prostate using the rover software (ABX GmbH, Radeberg, Germany), a 3D mask was placed around the volume of interest (VOI). Rover uses a specific algorithm that delineates the tumor automatically. This is achieved by adaptive thresholding and taking the background signal of the surrounding tissue into account [20]. In some cases, the strong radiotracer signal from the bladder was interfering with a fully automatic delineation of the tumor. Therefore, the tumor delineation was inspected in axial, coronal, and sagittal planes in all cases and the VOIs were corrected manually, if necessary. Computed parameters of the VOIs included the ASP, SUVmax, SUVmean, SUVpeak and the viable tumor volume (VTV). Figures 1 and 2 show examples of the delineation of the tumor using the rover software in patients with different Gleason scores (GSc).

### Viable tumor volume and tumor lesion binding rate

Accumulation of ^68^Ga-PSMA-HBED-CC in PC cells depends on the PSMA expression on the cell surface. PSMA is highly overexpressed in PC cells, resulting in a strong signal in PET [26]. The accumulation of ^68^Ga-PSMA-HBED-CC is reduced in non-diseased prostate tissue, expressing lower PSMA levels on their cell surface. This leads to a delineation of a VTV, at the location at which ^68^Ga-PSMA-HBED-CC internalization is highly active. Derived from the “total lesion glycolysis” used in ^18^F-FDG-PET/CT, a further parameter used in this study was the “total lesion binding” rate (TLB). The TLB was defined as the product of the SUVmean and the VTV of a PC lesion.

### Gleason score

All 37 patients underwent core needle biopsy of the prostate for a histopathological characterization of tumor malignancy. The GSc takes into account that malignancy signs such as cell size, nucleus size, nucleus to cytosol ratio, abnormal mitosis, and necrosis affect the grading. The final score is an addition of the most common found tumor grade and the highest found tumor grade for all tissue samples [27].

### TNM and D’Amico classification

TNM is a clinical used score for staging, for determination of prognosis and treatment planning. T-Stage describes the tumor size and tumor infiltration in surrounding tissue. N-stage indicates the presence of lymph node metastases; M-stage describes the presence of remote metastases outside the prostate [28].

The risk stratification for progression of PC can be measured with the D’Amico classification tool. It takes PSA blood level, clinical tumor size (through endorectal examination), and GSc (core needle biopsy) into account. It classifies patients according to low, intermediate, or high risk for early metastasis and increased tumor aggressiveness [29].

### Prostate imaging reporting and data system

Prostate imaging reporting and data system (PI-RADS) is a score used for standardized reporting of clinical findings in magnetic resonance imaging examinations of the prostate. In 2014, an update towards version 2 was released. MRI sequences included in PI-RADS v2 evaluation, which was used for all patients in this study, are high-resolution T2-weighted imaging, diffusion-weighted imaging, and dynamic contrast-enhanced imaging sequences. For each sequence, a score of 0–5 indicates the probability of a clinical significant PC lesion leading from improbable to highly suspicious in score 5. Every lesion is scored in the three sequences resulting in three sub-scores. The resulting final PI-RADS score is a summarized score [30, 31].

### Standardized uptake value

The SUV is a degree of tracer uptake in a specific region of interest (ROI) or VOI. It is calculated as the product of the activity concentration (Bq/g) and the patient’s weight (g) divided through the applied dose (Bq). SUVmax, SUVmean, and SUVpeak can be calculated for every ROI or VOI [32]. In addition to maximum and mean SUV, SUVpeak was computed as the mean value of a 3D sphere with a diameter of approximately 1.2 cm centered at the VOI maximum. All parameters were computed using the rover software.

### Statistical analysis

Descriptive statistics, correlations, and scatter plots were computed using MedCalc Statistical Software version 17.2 (MedCalc Software bvba, Ostend, Belgium; http://www.medcalc.org; 2017). All univariate correlations including ordinal variables were tested using Spearman’s rank correlation method. Pearson’s correlation method was used for metric variables. A *p* value < 0.05 was considered statistically significant. The polytomous universal model implemented in the statistic software IBM SPSS (version 24) was used for the ordinal logistic regression. Ordinal regression models the propensity of the first ranked state against all higher ranked states. This is repeated for the second and ongoing ranked states resulting in *k*–1 intercept parameters for *k* ordinal levels. We included the independent variables ASP, VTV, and TLB in the model. Ordinal regression is preferable, when the outcome consists of several discrete but ordered states instead of the assumption of a continuous dependent variable in linear regression. The group-based analysis for GSc was tested using Bonferroni corrected *t*-tests in SPSS. ASP probability was visualized using R software (Version 3.2.5, Vienna, Austria, http://www.R-project.org). Variables are reported as mean as well as standard deviation (SD).

## Results

### Association of asphericity with histopathology

**Fig. 3:**
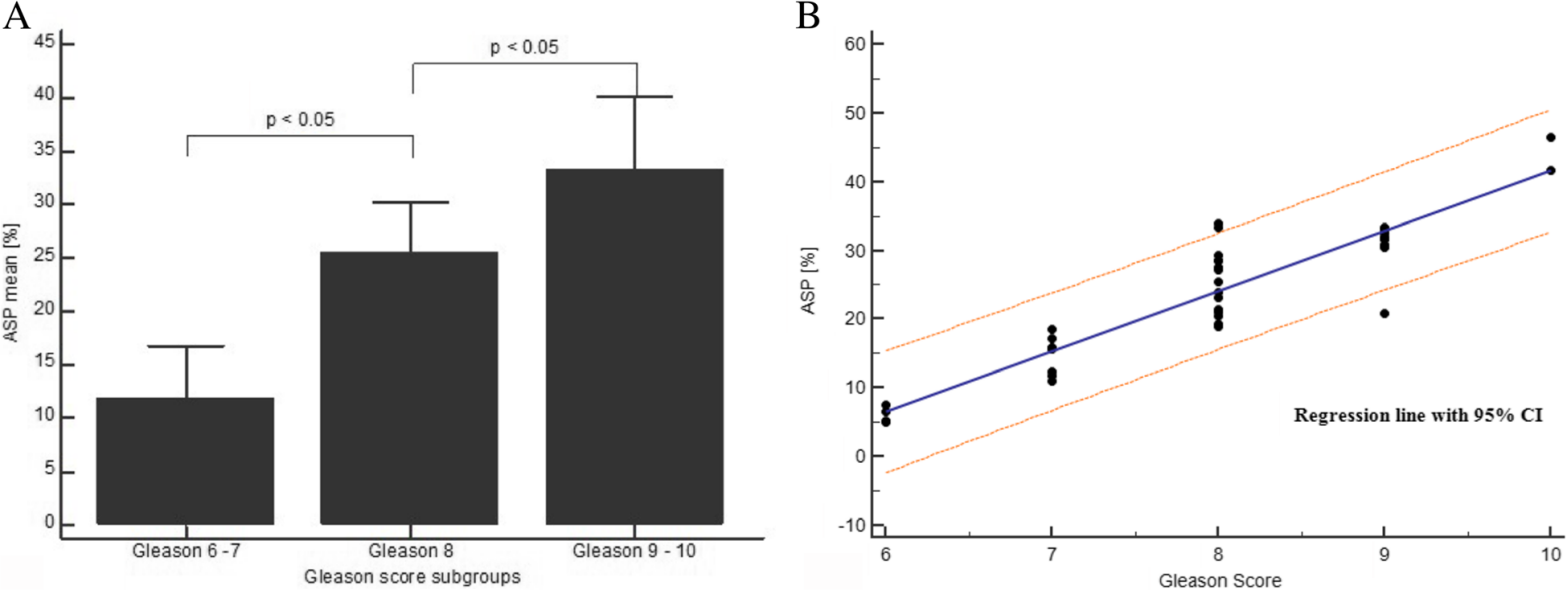
Differences between the asphericity for prostate cancer lesions with a Gleason score of 6–7, 8, and 9–10. **a** The bar chart demonstrates the asphericity values for the different Gleason score subgroups. A significant difference (*p* ≤ 0.05) was measured between prostate cancer lesions with a Gleason score of 6–7 and 8 and lesions with a Gleason score of 8 and 9–10. This potentially indicates that an increased tumor dedifferentiation/heterogeneity is accompanied by a gradual rise in the asphericity. Error bars indicate standard deviations. **b** The scatter diagram presents the correlation of the asphericity towards the Gleason scores. The linear regression line and the 95% confidence interval is shown. ASP: asphericity

Patients in this study demonstrated GSc ranging from 6 to 10 with a median of 8. Computing of ASP resulted in an overall mean 23.2 ± 10.1% ranging from 5 to 46.5% (CI 19.8–26.5). A close correlation was found between ASP and GSc (rho = 0.88; *p* < 0.05; CI 0.78–0.94). Twelve patients with GSc of 6–7 demonstrated an average ASP of 11.9 ± 4.8% (range 5.0–18.6%). Fifteen patients with GSc of 8 demonstrated an average ASP of 25.5 ± 4.8% (range 18.9–33.9%). Ten patients with GSc of 9–10 demonstrated an average ASP of 33.3 ± 6.8% (range 20.8–46.5%). Group-based analysis showed significant (*p* < 0.05) differences in ASP levels for Gleason 6–7 vs. Gleason 8 and for Gleason 8 vs. Gleason 9–10. The bar chart in Fig. 3 demonstrates the subgroup analysis. The scatter plot shows the regression with the associated 95% confidence interval. The correlation was shown to be significant (*R* ^2^ = 0.84, *p* < 0.05).

### Probability of Gleason score based on asphericity

**Fig. 4:**
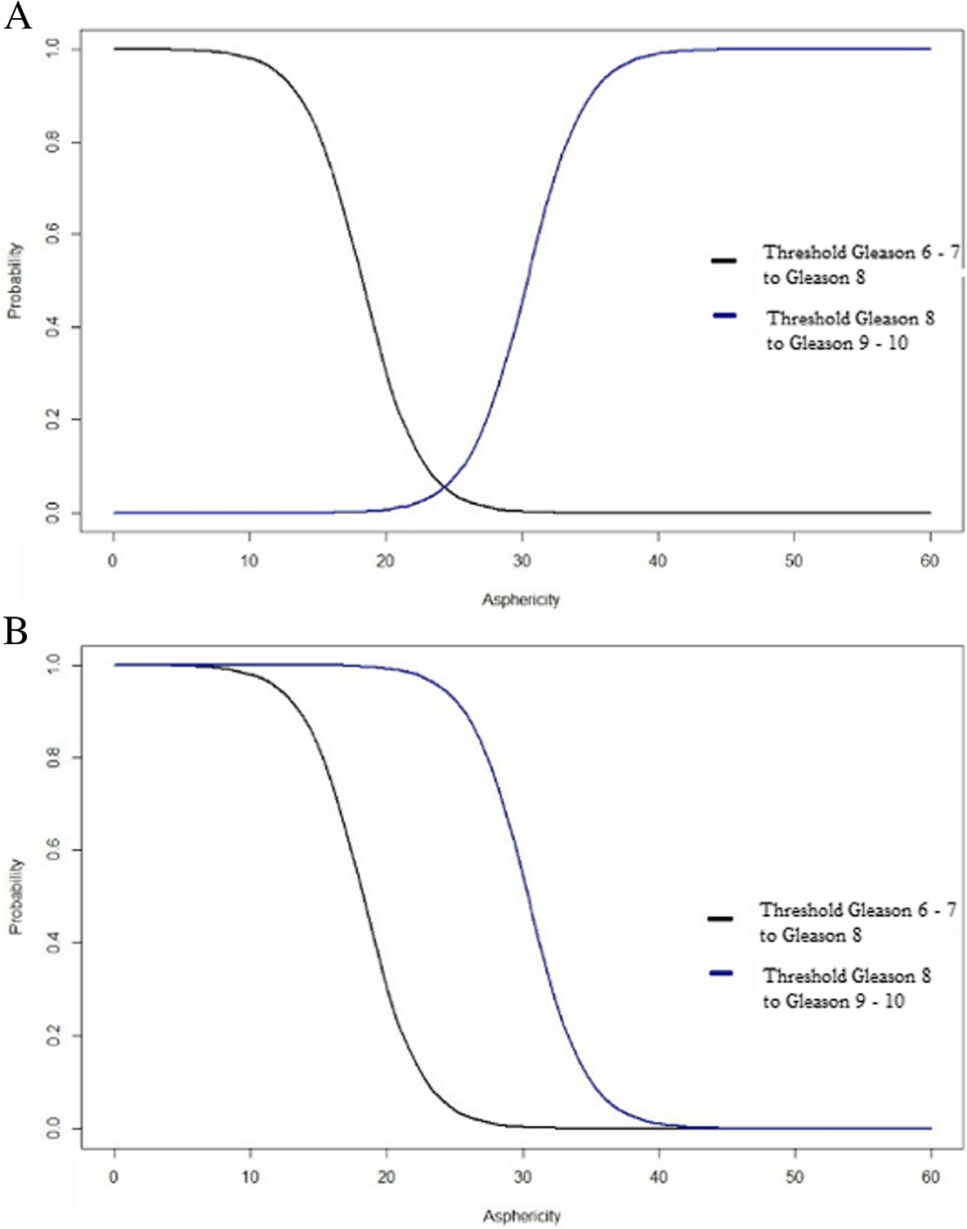
Probabilities of a Gleason score for different asphericity values. **a** The chart plot demonstrates the relative probability for the differentiation between prostate cancer lesions with Gleason score 6–7 and 8 as well as Gleason score 8 and 9–10. The area left of the black curve indicates the probability of Gleason 6–7, the area between black and blue curves indicate the probability of an ASP being scored Gleason 8 and the area to the right of the blue curve indicate the probability for Gleason 9–10. **b** The lower chart demonstrates cumulative probabilities of a Gleason score subgroup based on an ASP value containing the same areas beside the curves as described above

Ordinal logistic regression computed statistically significant (*p* < 0.05) effects to predict GSc based on given ASP. Threshold ASP values were computed, for the differentiation of the different GSc. Threshold values for a change from subgroup Gleason 6–7 to Gleason 8 was computed at an ASP of 5.6% (CI 7.3–29.2%) and probable change from subgroup Gleason 8 to Gleason 9–10 at an ASP of 30.4% (CI 13.2–48.9%). Grouped ranges were 0–5.6% for Gleason 6–7, 5.6–30.4% for Gleason 8 and ≥ 30.4% for Gleason 9–10. These results are summarized in Fig. 4.

### Association of asphericity with D’Amico classification, N-stage, and PI-RADS score

ASP and D’Amico classification showed a moderate correlation (rho = 0.6; *p* < 0.05; CI 0.32–0.78). Low risk presented an ASP of 5.0%, intermediate risk demonstrated an average ASP of 15.2 ± 2.1% (range 11.7–17.1%), and high risk presented an average ASP of 27.2 ± 8.5% (range 5.2–46.5%). No significant (*p* > 0.05) correlation was measured between ASP and N-stage (rho = − 0.07; *p* > 0.05; CI − 0.51–0.4). Additionally, a non-significant (*p* > 0.05) weak correlation was seen between ASP and the PI-RADS score (rho = 0.26; *p* > 0.05; CI − 0.11–0.57). All results are summarized in Table 2.

**Table 2:** Summary of correlations between in vivo measurements on 68Ga-PSMA PET and ex vivo parameters

		GSC	D’Amico classification	N-stage	PI-RADS score
ASP (%)	Rho	*0.88*	*0.6*	− 0.07	0.26
	*p* value	*p < 0.05*	*p < 0.05*	*p* > 0.05	*p* > 0.05
	CI 95%	*0.78–0.94*	*0.32–0.78*	− 0.51–0.4	− 0.11–0.57
VTV (cm^3^)	Rho	*0.51*	*0.49*	− 0.06	0.33
	*p* value	*p < 0.05*	*p < 0.05*	*p* > 0.05	*p* > 0.05
	CI 95%	*0.23–0.72*	*0.17–0.71*	− 0.5–0.4	− 0.04–0.62
TLB	Rho	*0.43*	*0.38*	0	0.3
	*p* value	*p < 0.05*	*p < 0.05*	*p* > 0.05	*p* > 0.05
	CI 95%	*0.12–0.66*	*0.04–0.64*	− 0.45–0.45	− 0.07–0.6
SUVmax	Rho	0.29	0.29	− 0.14	0.22
	*p* value	*p* > 0.05	*p* > 0.05	*p* > 0.05	*p* > 0.05
	CI 95%	− 0.04–0.56	− 0.06–0.58	− 0.56–0.33	− 0.15–0.54
SUVmean	Rho	0.24	0.2	0.13	0.1
	*p* value	*p* > 0.05	*p* > 0.05	*p* > 0.05	*p* > 0.05
	CI 95%	− 0.09–0.53	− 0.15–0.51	− 0.35–0.55	− 0.27–0.44
SUVpeak	Rho	0.3	0.3	− 0.05	0.13
	*p* value	*p* > 0.05	*p* > 0.05	*p* > 0.05	*p* > 0.05
	CI 95%	− 0.03–0.57	− 0.05–0.58	− 0.49–0.42	− 0.24–0.47
PSA (ng/ml)	Rho	0.27	0.15	0.07	0.12
	*p* value	*p* > 0.05	*p* > 0.05	*p* > 0.05	*p* > 0.05
	CI 95%	− 0.06–0.55	− 0.21–0.47	− 0.4–0.51	− 0.25–0.46

Spearman’s rank correlation method. This table summarizes the associations between the ASP, VTV, TLB, SUVmax, SUVmean, SUVpeak and PSA in the left column and the GSc, D’Amico classification, N-stage and the PI-RADS score in the upper row using Spearman’s rank correlation method. The closest significant (*p* < 0.05) correlations were measured between ASP and GSc followed by a moderate correlation towards the D’Amico classification. A significant (*p* < 0.05) weak correlation was seen between VTV and the GSc. Statistically significant correlations (*p* < 0.05) are highlighted in italics. *Abbreviations: ASP* asphericity, *VTV* viable tumor volume, *TLB* total lesion binding rate, *SUVmax* maximum standardized uptake value, *SUVmean* mean standardized uptake value, *SUVpeak* peak standardized uptake value, *PSA* prostate-specific antigen blood level, *GSc* Gleason score, *PI-RADS* prostate imaging reporting and data system

### Association of viable tumor volume with Gleason score, D’Amico classification, N-stage, and PI-RADS score

The study cohort presented an average VTV of 12.3 ± 11.3 cm^3^ ranging from 0.8 to 54.1 cm^3^ and a moderate correlation could be demonstrated for VTV and GSc (rho = 0.51; *p* < 0.05; CI 0.23–0.72). VTV averages for GSc 6 were 6.1 ± 7.6 cm^3^ (range 0.8–17.3 cm^3^); for GSc 7, 7.5 ± 6.8 cm^3^ (range 2.3–23.6 cm^3^); for GSc 8, 11.4 ± 12.5 cm^3^ (range 2.9–54.1 cm^3^); for GSc 9, 23.0 ± 9.0 cm^3^ (range 10.8–39.1 cm^3^); and for GSc 10, 7.8 ± 6.7 cm^3^ (range 3–12.5 cm^3^). VTV presented a moderate association to the respective D’Amico classification (rho = 0.49; *p* < 0.05; CI 0.17–0.71). VTV was 3.9 cm^3^ for low risk; intermediate risk scored patients showed an average VTV of 4.7 ± 2.5 cm^3^ (range 2.3–8.9 cm^3^). Patients scored with high risk of progression demonstrated an average VTV of 14.6 ± 11.9 cm^3^ (range 2.9–54.1 cm). No significant (*p* > 0.05) correlation was found for VTV to N-stage (rho = − 0.06; *p* > 0.05; CI − 0.5–0.4). VTV presented a non-significant (*p* > 0.05) weak correlation to the PI-RADS score (rho = 0.33; *p* > 0.05; CI − 0.04–0.62). All results are summarized in Table 2.

### Association of total lesion binding rate with Gleason score, D’Amico classification, N-stage, and PI-RADS score

TLB presented an average of 99.2 ± 136.3 ranging between 3 and 584.3 showing statistical significant (*p* < 0.05) weak correlations to GSc (rho = 0.43; *p* < 0.05; CI 0.12–0.66) and to D’Amico classification (rho = 0.38; *p* < 0.05; CI 0.04–0.64). No correlation was found towards N-stage (rho = 0; *p* > 0.05; CI − 0.45–0.45) and a non-significant (*p* < 0.05) weak correlation was seen for TLB to the PI-RADS score (rho = 0.3; *p* > 0.05; CI − 0.07–0.6). All results are summarized in Table 2.

### Association of maximum, peak and mean standardized uptake values with Gleason score, D’Amico classification, N-stage, and PI-RADS score

SUVmax of all PC lesions presented an average of 18.1 ± 19.4 ranging between 3.3 and 83.2 and demonstrated non-significant (*p* > 0.05) weak correlations to GSc (rho = 0.29; *p >* 0.05; CI − 0.04–0.56) and D’Amico classification (rho = 0.29; *p* > 0.05; CI − 0.06–0.58). A non-significant (*p* > 0.05) very weak negative association was seen for SUVmax to N-stage (rho = − 0.14; *p* > 0.05; CI − 0.56–0.33) and a non-significant (*p* > 0.05) weak correlation towards PI-RADS score (rho = 0.22; *p* > 0.05; CI − 0.15–0.54). With an average of 6.1 ± 3.2 ranging between 2.2 and 16.8, SUVmean presented comparable non-significant (*p* > 0.05) weak to very weak correlations to GSc (rho = 0.24; *p >* 0.05; CI − 0.09–0.53), D’Amico classification (rho = 0.2; *p >* 0.05; CI − 0.15–0.51), N-stage (rho = 0.13; *p >* 0.05; CI − 0.35–0.55), and PI-RADS score (rho = 0.1; *p* > 0.05; CI − 0.27–0.44). SUVpeak presented an average of 15.3 ± 15.9 ranging between 3.1 and 76.4 with non-significant (*p* > 0.05) weak correlations to GSc (rho = 0.3; *p* > 0.05; CI − 0.03–0.57) and D’Amico classification (rho = 0.3; *p* > 0.05; CI − 0.05–0.58). No correlation was seen towards N-stage (rho = − 0.05; *p* > 0.05; CI − 0.49–0.42) and a non-significant (*p >* 0.05) very weak correlation to PI-RADS score (rho = 0.13; *p >* 0.05; CI − 0.24–0.47). All results are summarized in Table 2.

### Association of prostate-specific antigen blood level with Gleason score, D’Amico classification, N-stage, and PI-RADS score

PSA blood samples were taken in mean 16.2 ± 25.6 days ranging 0 to 84 days and demonstrated an average of 17.7 ± 21.5 ng/ml ranging 0.23 to 116 ng/ml. PSA showed non-significant (*p >* 0.05) weak to very weak correlations to GSc (rho = 0.27; *p >* 0.05; CI − 0.06–0.55) and to D’Amico classification (rho = 0.15; *p >* 0.05; CI − 0.21–0.47). Additionally, no correlation was seen for PSA to N-stage (rho = 0.07; *p* > 0.05; CI − 0.4–0.51) and a weak non-significant (*p* > 0.05) correlation to PI-RADS score (rho = 0.12; *p >* 0.05; CI − 0.25–0.46). All results are summarized in Table 2.

#### Prognostic estimation of Gleason scores using asphericity, viable tumor volume, and total lesion binding rate on multivariable analysis

On multivariable analysis regarding independent association of ASP, VTV, and TLB with the GSc, the ASP and the VTV represented independent predictive parameters (0.71; CI 0.35–1.06 and 0.36; CI 0.03–0.69). The TLB did not represent an independent parameter (− 0.02; CI − 0.04–0.004). All variables are summarized in Table 3.

**Table 3:** Prognostic estimation of Gleason scores using asphericity, viable tumor volume, and total lesion binding rate on multivariable analysis

	Estimate	*p* value	Confidence interval 95%
ASP (%)	0.71	*p* < 0.05	0.35–1.06
VTV (cm^3^)	0.36	*p* < 0.05	0.03–0.69
TLB	− 0.02	*p* > 0.05	− 0.04–0.004

On multivariable analysis using ordinal logistic regression, correlation of the ASP to GSc was independent against VTV and TLB. A comparable weaker effect was found for VTV as well. *Abbreviations: ASP* asphericity, *VTV* viable tumor volume, *TLB* total lesion binding rate

#### Association of maximum, peak and mean standardized uptake values and prostate-specific antigen blood level with asphericity, viable tumor volume, and total lesion binding rate

SUVmax demonstrated a weak non-significant (*p* > 0.05) correlation to the ASP (rho = 0.23; *p* > 0.05; CI − 0.1–0.51) and statistically significant (*p* < 0.05) moderate correlations to VTV (rho = 0.58; *p* < 0.05; CI 0.32–0.76) and TLB (rho = 0.71; *p* < 0.05; CI 0.5–0.84). As expected, SUVmean and SUVpeak did show comparable associations as seen for SUVmax. SUVmean presented a non-significant (*p >* 0.05) weak correlation to the ASP (rho = 0.17; *p >* 0.05; CI − 0.17–0.47), and moderate correlations to VTV (rho = 0.68; *p* < 0.05; CI 0.46–0.83) and TLB (rho = 0.84; *p* < 0.05; CI 0.7–0.91). SUVpeak showed a non-significant (*p >* 0.05) weak correlation to the ASP (rho = 0.15; *p >* 0.05; CI − 0.19–0.45) followed by moderate correlations to VTV (rho = 0.62; *p* < 0.05; CI 0.38–0.79) and TLB (rho = 0.77; *p* < 0.05; CI 0.59–0.88). PSA blood level showed non-significant (*p >* 0.05) weak correlations to ASP (rho = 0.25; *p >* 0.05; CI − 0.08–0.53), VTV (rho = 0.32; *p >* 0.05; CI − 0.04–0.58), and TLB (rho = 0.22; *p >* 0.05; CI − 0.11–0.51). Table 4 summarizes these results.

**Table 4:** Summary of correlations between asphericity, viable tumor volume derived from 68Ga-PSMA-PET, TLB, SUVmax, SUVmean, SUVpeak and the PSA value

		ASP (%)	VTV (cm^3^)	TLB
SUVmax	Rho	0.23	0.58	0.71
	*p* value	*p* > 0.05	*p* < 0.05	*p* < 0.05
	CI 95%	− 0.1–0.51	0.32–0.76	0.5–0.84
SUVmean	Rho	0.17	0.68	0.84
	*p* value	*p* > 0.05	*p* < 0.05	*p* < 0.05
	CI 95%	− 0.17–0.47	0.46–0.83	0.7–0.91
SUVpeak	Rho	0.15	0.62	0.77
	*p* value	*p* > 0.05	*p* < 0.05	*p* < 0.05
	CI 95%	− 0.19–0.45	0.38–0.79	0.59–0.88
PSA (ng/ml)	Rho	0.25	0.32	0.22
	*p* value	*p* > 0.05	*p* > 0.05	*p* > 0.05
	CI 95%	− 0.08–0.53	− 0.04–0.58	− 0.11–0.51

Pearson’s correlation method. This table summarizes the associations between the ASP, VTV, TLB, SUVmax, SUVmean, SUVpeak and PSA blood level using Pearson’s correlation method. Neither of the investigated associations presented a significant correlation (*p* > 0.05) to the ASP. Significant (*p* < 0.05) moderate correlations were demonstrated for VTV and TLB to SUVmax and as expected also to SUVmean and SUVpeak. PSA did not show a significant correlation (*p* > 0.05) to the investigated parameters. *Abbreviations: ASP* asphericity, *VTV* viable tumor volume, *TLB* total lesion binding rate, *SUVmax* maximum standardized uptake value, *SUVmean* mean standardized uptake value, *SUVpeak* peak standardized uptake value, *PSA* prostate-specific antigen blood level

## Discussion

This study demonstrated that the ASP in ^68^Ga-PSMA-PET could represent a promising quantitative parameter for an improved non-invasive T-staging of patients with PC. In the investigated patient collective, patient groups with different GSc could be discriminated based on the quantitative assessment of the ASP of the local PC. On multivariable analysis, it was demonstrated that the ASP was independently associated with the GSc.

### Asphericity for the evaluation of tumor heterogeneity

Aggressiveness of tumor behavior, therapy response and overall patient survival is known to be associated with the heterogeneity of the tumor [33, 34]. The parameter ASP enables the quantification of the associated spatial irregularities in PET datasets. Previous studies already investigated the prognostic value of the ASP in certain types of head and neck cancers and non-small-cell lung cancer (NSCLC) [15, 19, 21, 35]. The ASP was found to be an independent predictor of outcome in head and neck cancer patients undergoing pre-therapeutic ^18^F-FDG-PET/CT. ASP measurements of the ^18^F-FDG uptake also improved the prediction of tumor progression. These previous studies reported a moderate correlation between ASP and MTV, while no correlation between ASP and SUVmax were measured [19, 35]. Additionally, a recent study demonstrated a significant association between progression-free survival and overall survival based on the assessment of the ASP [15].

Comparable results were published for NSCLC, in which the ASP provided a higher prognostic value for progression-free survival and overall survival in NSCLC patients compared to SUVmax, MTV, and another parameter of spatial heterogeneity called solidity [19]. Moderate associations were found between ASP and MTV; no correlation was measured between SUVmax and ASP [19]. Additionally, correlations of ASP with histopathology and with the expression of the tumor proliferation markers KI-67 and epidermal growth factor receptor (EGFR) in NSCLC were found [35].

These previous studies introduced the ASP as a promising novel parameter for the non-invasive characterization of tumors. Additionally, the ASP could represent a strong predictive parameter regarding the overall survival in these patient collectives [15, 19].

### Evaluation and local staging of prostate cancer by positron emission tomography

Various radiotracers have been tested for the local staging of PC. One of these tracers is ^18^F-FDG. Its accumulation is based on an increased glucose metabolism of cancer cells due to an overexpression of hexokinase. ^18^F-FDG uptake was shown to be increased in benign prostate tissue, including prostate hyperplasia, as well as in PC cells [11]. Sensitivities of up to 64% for detection of primary PC were reported [36]. The limited performance of ^18^F-FDG for the primary PC diagnosis could be associated with the relatively low metabolic rate of PC and the lack of patient selection in these previous studies [6, 8–10, 37].

A different tracer that demonstrated potential for the detection of PC is ^18^F-C. The use of choline-based tracers is dependent on phosphorylcholine turnover in PC cells. Most studies, however, reported limited sensitivities, especially for the primary diagnosis of PC [6, 7, 11]. A further tracer that was evaluated in this context is ^11^C-acetate. Its uptake is a result of an increased lipid synthesis in tumor cells [38]. Even though its uptake is not limited to PC cells, this radiotracer was shown to be superior to ^18^F-FDG for the detection of PC lesions [39]. ^11^C-MET and ^18^F-FDHT have also been evaluated for the staging of PC. ^11^C-MET targets the increased amino-acid transport of methionine for protein synthesis in cancer cells. ^18^F-FDHT targeting is based on an overexpression of the androgen receptor. Limitations of these tracers include the lack of studies regarding their diagnostic value [6].

These previous studies demonstrated that novel more specific tracers and in vivo parameters are needed for an improved in vivo detection and characterization of PC.

### ^68^Ga-PSMA-PET for the staging of prostate cancer lesions

PSMA is significantly over-expressed in PC cells and over-expression increases with more advanced tumor stages [26]. Binding of ^68^Ga-PSMA-HBED-CC leads to receptor internalization and tracer accumulation. It is important to mention that PSMA avid tissue can be found throughout the body since it is a zinc-dependent exopeptidase with glutamate carboxypeptidase activity [24]. A recent study demonstrated promising sensitivity, specificity, and accuracy rates of 65.9, 98.9, and 88.5% for the detection of high-risk PC using ^68^Ga-PSMA-HBED-CC. However, a reliable detection can be challenging, as up to 8.4% of all primary tumors showed no tracer accumulation [13, 40]. Another recent study investigated the intensity of tracer accumulation in 90 patients using ^68^Ga-PSMA-HBED-CC. It was demonstrated that the SUVmax of primary PC was significantly higher in GSc > 7 compared to GSc < 7. Additionally, it was shown that a PSA value > 10 ng/ml was an associated with a significantly higher tracer uptake compared to a PSA value < 10 ng/ml [41]. Other studies focused on the diagnostic accuracy of recurrent PC using ^68^Ga-PSMA-HBED-CC. These studies reported sensitivities and specificities up to 80 and 97% for the detection of recurrent PC [42–45]. PSA blood levels in biochemical recurrence correlated with positive findings, even in patients with low PSA levels (< 1 ng/ml) [14, 43, 46].

### Potential of asphericity as a novel diagnostic parameter in the staging process of patients with ^68^Ga-PSMA-PET-positive prostate cancer lesions

To the best of our knowledge this was the first study which combined ^68^Ga-PSMA-PET with the evaluation of the ASP in patients with ^68^Ga-PSMA-HBED-CC-positive PC lesions. Previous studies have, as described earlier, focused on the investigation of the ASP in combination with other radiotracers, such as ^18^F-FDG-PET.

The current study demonstrated that the ASP derived from ^68^Ga-PSMA-PET enables a distinction between patient groups with different GSc. Additionally, a correlation of the ASP with the GSc, based on core needle biopsy, was found. The findings in our studies are in line with previous studies, in which a correlation between the ASP and the histopathological staging of NSCLC was demonstrated [35]. In the current study, no significant correlation between SUVmax and ASP was measured [15, 19, 35]. Furthermore, no significant correlation was found between SUVmax and GSc. In contrast to previous studies, this study did not demonstrate a correlation of ASP with the PET tumor volume. This could be explained by smaller VTV of PC lesions in comparison to lesions of NSCLC and head and neck cancer. Our study did not show statistically significant associations of the ASP to the PI-RADS score.

Further prospective studies and a higher number of patients are now warranted to investigate the potential of the ASP in the staging process of PC patients.

This study is limited by its retrospective study design. Only a relatively small patient cohort with clustered GSc was investigated. In case of multiple lesions, PC lesion selection on PET was based on the evaluation of the MRI data. If the strong radiotracer signal from the bladder was interfering with an automatic delineation of the tumor, the tumor delineation was inspected in axial, coronal, and sagittal planes and VOIs were corrected manually, if necessary. Although several viable automated algorithms have been described, the VTV is presently still determined by manual delineation in a high number of institutions [26, 47–54]. Manual delineation is prone to intra- and interobserver variability as well as to potentially size- and background-dependent bias if fixed absolute or relative thresholds are used.

## Conclusions

The ASP in ^68^Ga-PSMA-PET could represent a promising parameter for an improved non-invasive T-staging of patients with PC. Further prospective studies are now warranted to investigate the potential of the ASP in the staging process of PC patients.

## Abbreviations

^18^F-FDHT: ^18^F-fluorodihydrotestosterone
^11^C-MET: 1-S-methyl-^11^C-methionine
^18^F-FDG: 2-deoxy-2-^18^F-fluoro-D-glucose
^11^C-C: N-methyl-^11^C-choline
^18^F-C: ^18^F-fluoromethyl-[1,2-2H4]choline
^68^Ga-PSMA-HBED-CC: “Glue-NH-CO-NH-Lys” radio-labeled with ^68^Ga-N,N-bis[2-hydroxy-5-(carboxyethyl)benzyl]-ethylendiamine-N,N diacetic acid
^68^Ga-PSMA-PET/CT: ^68^Ga-PSMA-HBED-CC-based positron emission tomography fused with computed tomography
ASP: Asphericity
CI 95%: 95% Confidence interval
GSc: Gleason score (core needle biopsy)
MRI: Magnetic resonance imaging
MTV: Metabolic tumor volume
NSCLC: Non-small cell lung cancer
PC: Prostate cancer
PET: Positron emission tomography
PET/CT: Positron emission tomography fused with computed tomography
PI-RADS: Prostate imaging reporting and data system
PSMA: Prostate-specific membrane antigen
ROI: Region of interest
SUV: Standardized uptake value
TLB: Tumor lesion binding rate
VOI: Volume of interest
VTV: Viable tumor volume
